# Supplementary material for: Typical neural adaptation for familiar images in autistic adolescents
Source: Imaging Neurosci (Camb). 2025 Mar 19;3:imag_a_00505. doi: 10.1162/imag_a_00505 (PMC12319762; doi:10.1162/imag_a_00505)
Supplement: Supplementary Material [file imag_a_00505-supp.pdf]

Supplementary material

for:

Typical neural adaptation for familiar images in autistic adolescents

Britta U. Westner*, Ella Bosch*, Christian Utzerath, Jan Buitelaar, Floris P. de Lange
*these authors contributed equally.

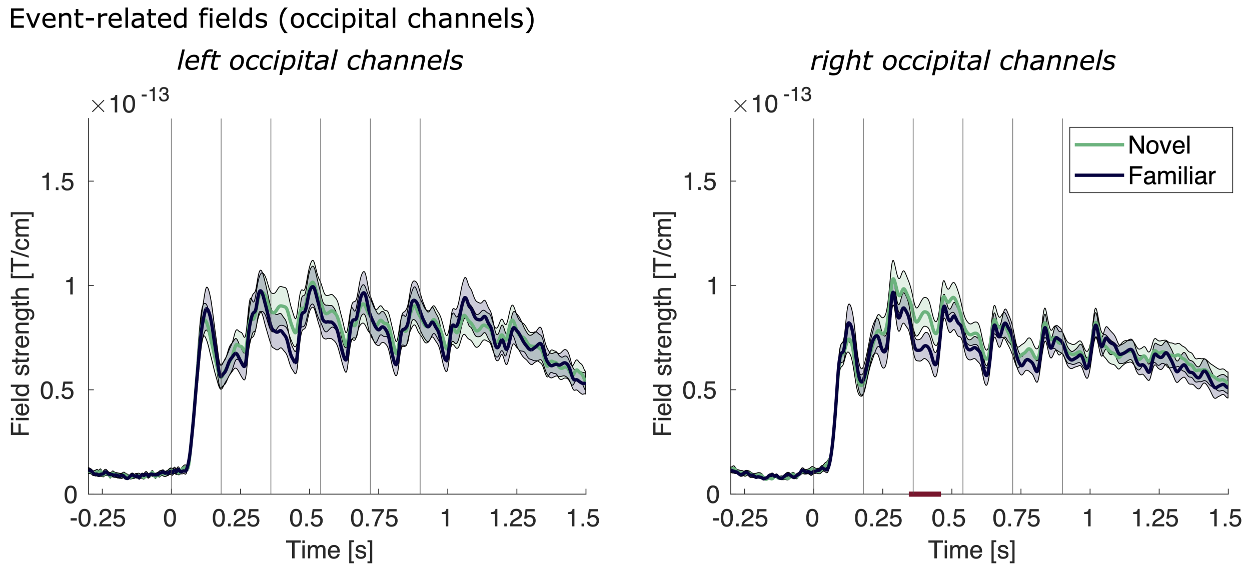

Figure S1. Event-related fields of the left occipital channel group (left) and right occipital channel group (right) following image presentation for the student group, averaged across participants. The familiar condition is shown in dark blue, the novel condition in green. Shaded areas mark the SEM across participants. Vertical lines mark the image onsets. The bars mark the clusters across time from a cluster permutation test ($p<0.05$).

Sensitivity analyses

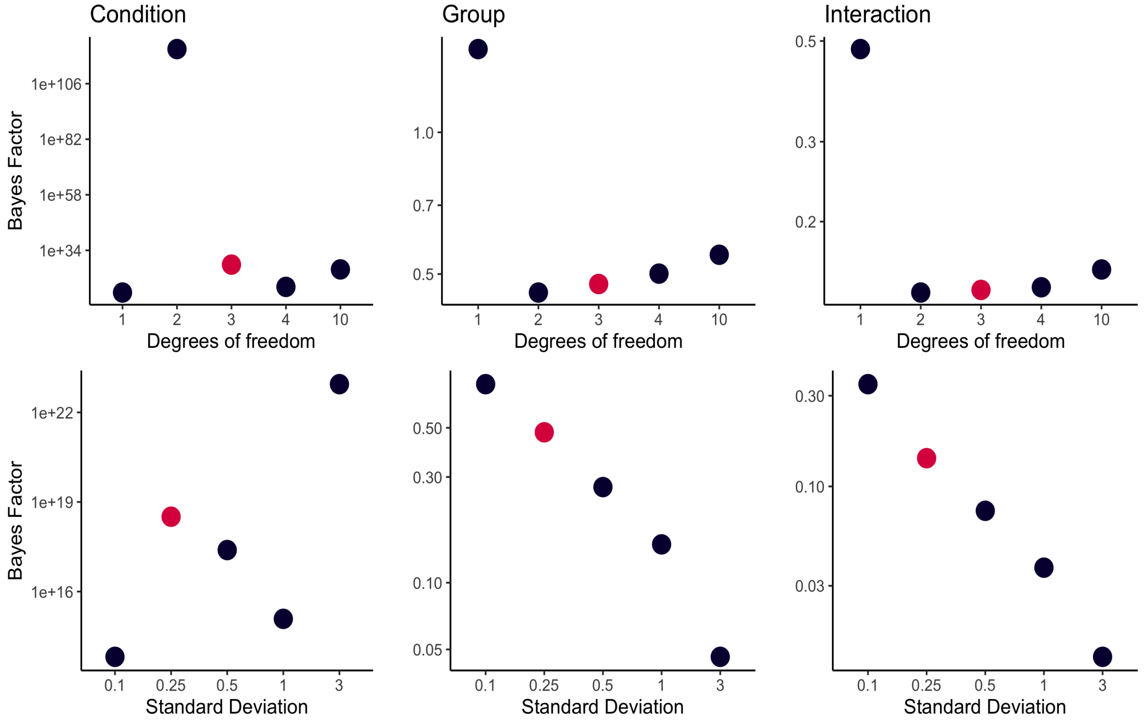

Figure S2. Sensitivity analysis for **percentage correct**, varying the shape of the priors. The upper row shows the Bayes Factors per parameter (*condition*, *group*, and the interaction *condition* × *group*), keeping the mean and standard deviation fixed but varying the degrees of freedom (df = [1, 2, 3, 4, 10]). The lower row shows the Bayes Factors per parameter, keeping the degrees of freedom and mean fixed, but varying the standard deviation (σ = [0.1, 0.25, 0.5, 1.0, 3.0]). The pink dot shows the degrees of freedom and standard deviation used in the model.

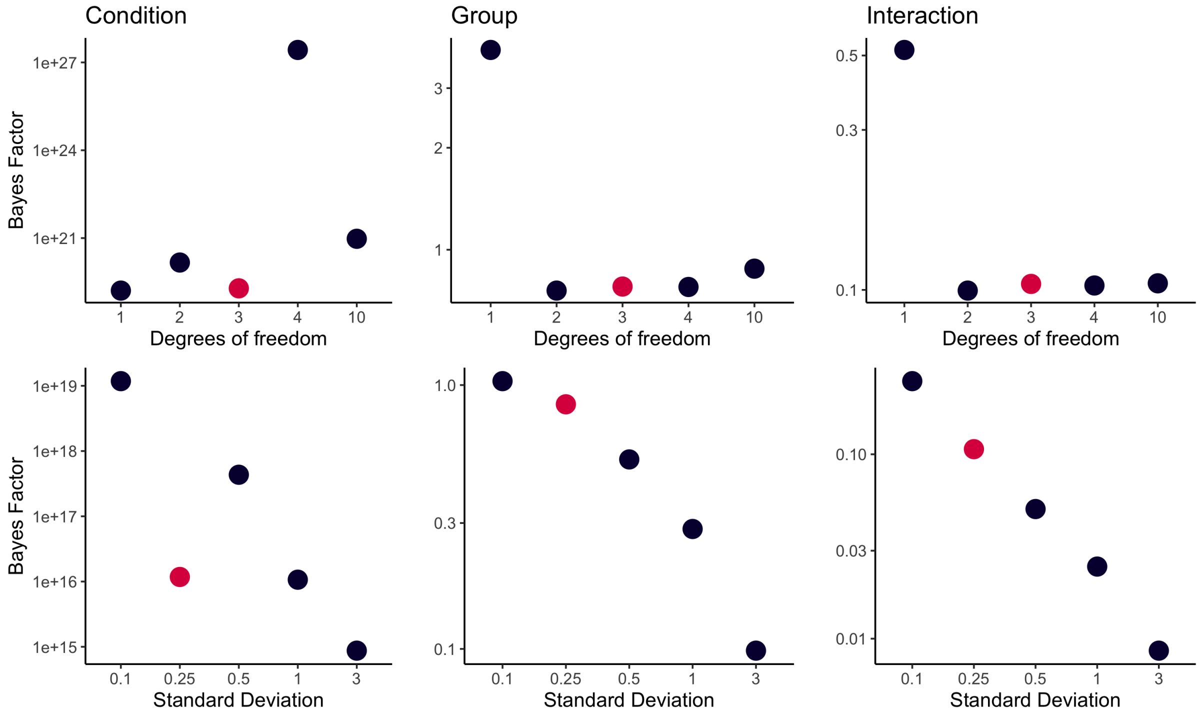

Figure S3. Sensitivity analysis for **reaction time**, varying the shape of the priors. Layout of the figure as in Figure S2.

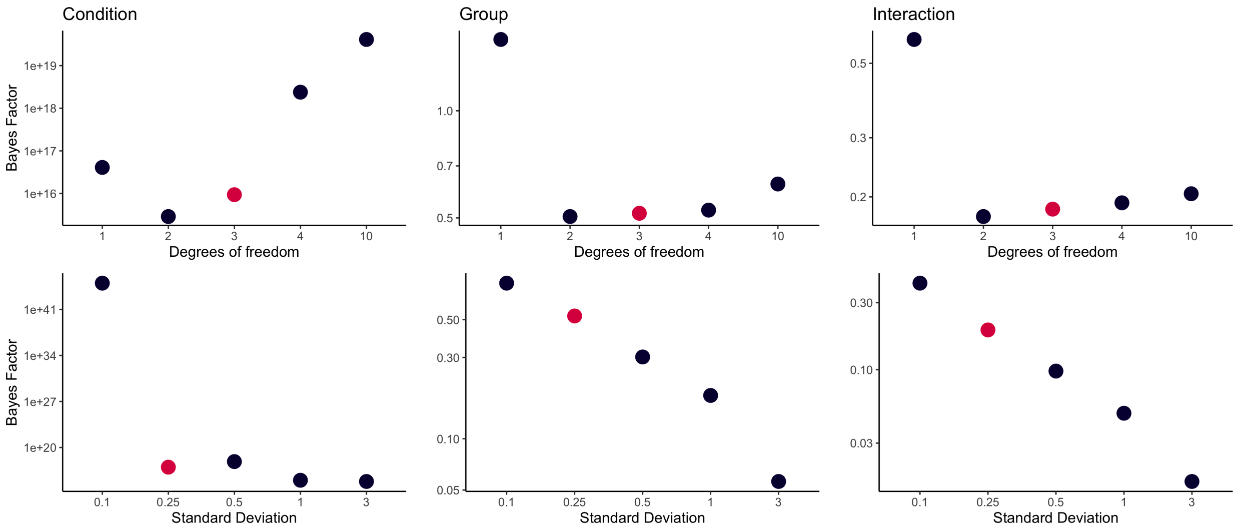

Figure S4. Sensitivity analysis for **event-related fields amplitude**, varying the shape of the priors. Layout of the figure as in Figure S2.

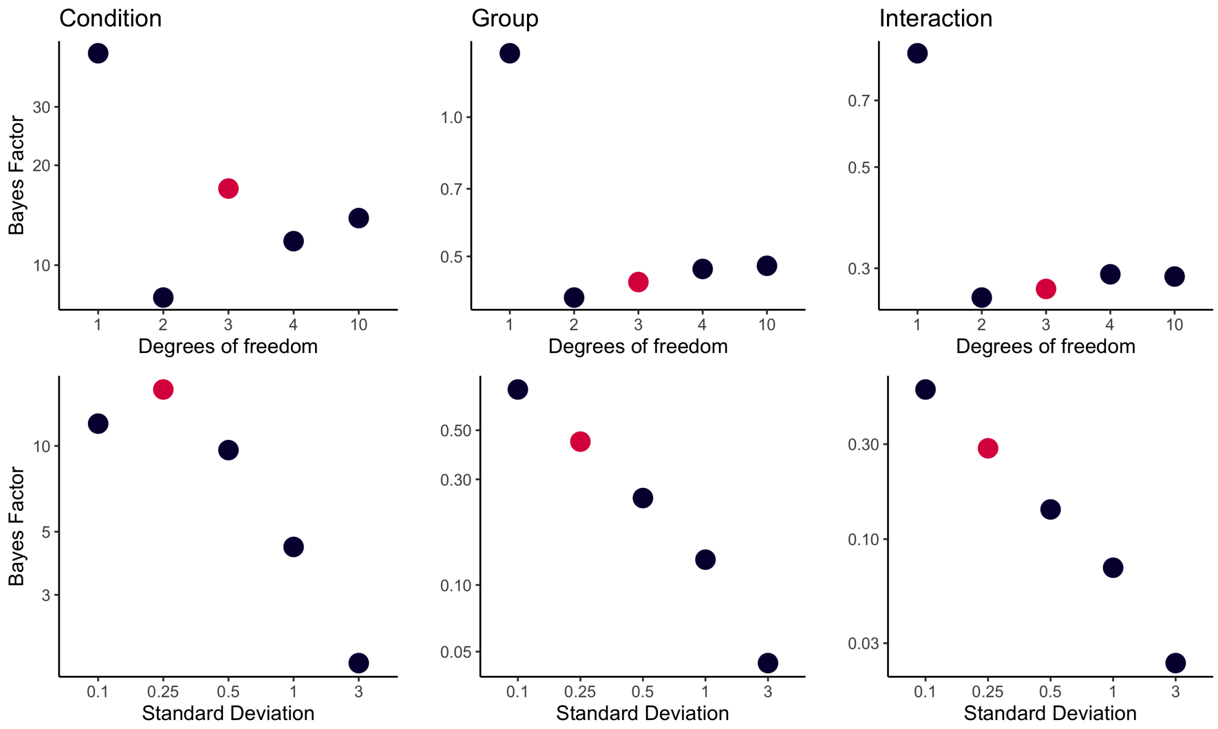

Figure S5. Sensitivity analysis for **entrainment power**, varying the shape of the priors. Layout of the figure as in Figure S2.

Bayesian Mixed Effects Models with Group

	Estimate	Est. Error	95% CI	BF_{10}
Intercept	0.00	0.16	-0.31, 0.31	
Condition	-0.19	0.03	-0.26, -0.13	**>1000**
Group	-0.01	0.17	-0.32, 0.34	**0.0623**
Cond × Group	-0.02	0.03	-0.09, 0.04	**0.0149**

Table S1: Model output **percentage correct** with **Group**.
R^2: 0.9172, Est. Error: 0.0176

	Estimate	Est. Error	95% CI	BF_{10}
Intercept	0.00	0.17	-0.33, 0.33	
Condition	0.23	0.03	0.17, 0.29	**>1000**
Group	0.20	0.17	-0.13, 0.54	0.1383
Cond × Group	0.00	0.03	-0.06, 0.06	**0.0108**

Table S2: Model output **response times** with **Group**.
R^2: 0.9438, Est. Error: 0.0115

	Estimate	Est. Error	95% CI	BF_{10}
Intercept	-0.01	0.16	-0.31, 0.31	
Condition	0.37	0.05	0.27, 0.47	**>1000**
Group	0.10	0.15	-0.19, 0.40	**0.0701**
Cond × Group	0.02	0.05	-0.08, 0.12	**0.0206**

Table S3: Model output **event-related fields amplitude** (0.208-0.713 s, right temporal channels) with **Group**. R^2: 0.8152, Est. Error: 0.0399

	Estimate	Est. Error	95% CI	BF_{10}
Intercept	-0.09	0.13	-0.35, 0.18	
Condition	-0.18	0.06	-0.29, -0.06	1.6263
Group	-0.06	0.13	-0.33, 0.20	**0.0543**
Cond × Group	0.05	0.06	-0.07, 0.17	**0.0306**

Table S4: Model output **entrainment power** (5.556 Hz, left occipital channels) with **Group.**
R^2: 0.5475, Est. Error: 0.0816

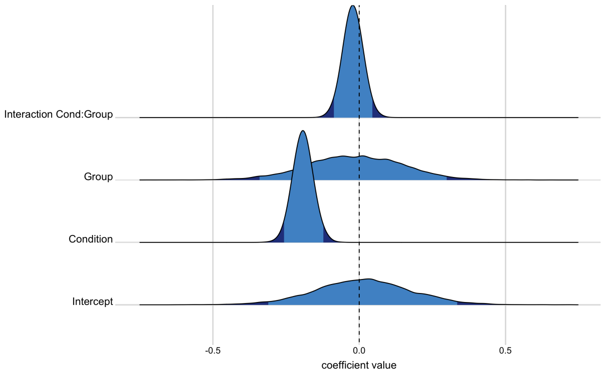

Figure S6. Posterior distributions for model of **percentage correct.** *For all posterior distribution figures: dark blue shading in the tails signifies the highest and lowest 2.5% of the distribution. Mid-blue shade represents the center 95%.*

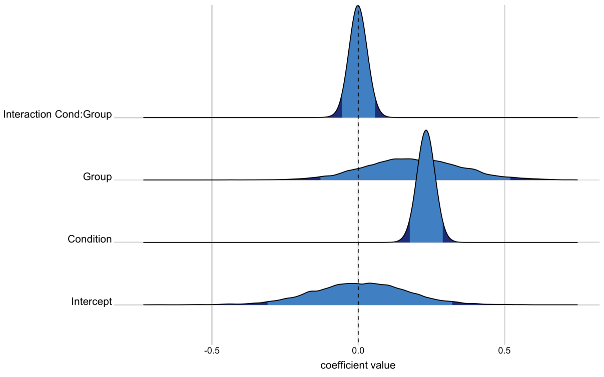

Figure S7. Posterior distributions for model of **reaction time.**

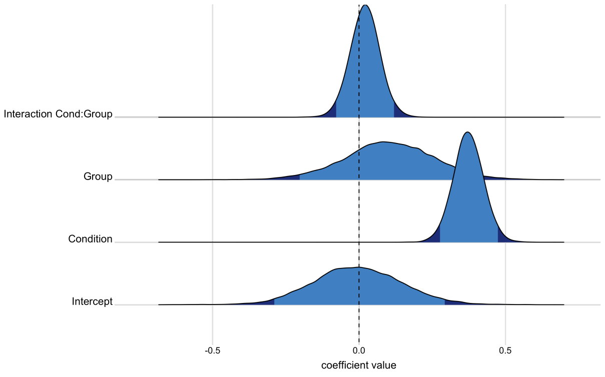

Figure S8. Posterior distributions for model of **event-related fields amplitudes.**

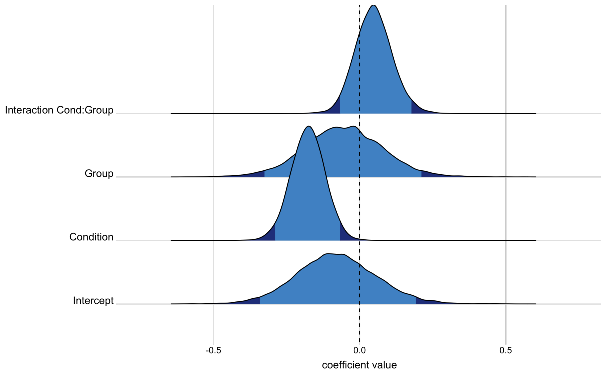

Figure S9. Posterior distributions for model of **entrainment power.**

Bayesian Mixed Effects Models with AASP

	Estimate	Est. Error	95% CI	BF_{10}
Intercept	-0.03	0.27	-0.55, 0.52	
Condition	-0.22	0.05	-0.32, -0.12	**28.847**
AASP	-0.26	0.27	-0.80, 0.26	0.1666
Cond × AASP	-0.02	0.05	-0.12, 0.09	**0.0202**

Table S5: Model output **percentage correct** with **AASP**.
R^2: 0.9239, Est. Error: 0.0270

	Estimate	Est. Error	95% CI	BF_{10}
Intercept	0.02	0.21	-0.41, 0.44	
Condition	0.24	0.05	0.14, 0.33	**>1000**
AASP	0.63	0.21	0.21, 1.03	6.4017
Cond × AASP	0.00	0.05	-0.10, 0.10	**0.0173**

Table S6: Model output **response times** with **AASP**.
R^2: 0.9438, Est. Error: 0.0224

	Estimate	Est. Error	95% CI	BF_{10}
Intercept	-0.07	0.23	-0.52, 0.38	
Condition	0.35	0.09	0.18, 0.53	**34.64**
AASP	-0.29	0.23	-0.75, 0.14	0.1808
Cond × AASP	-0.10	0.08	-0.28, 0.07	**0.0643**

Table S7: Model output **event-related fields amplitude** (0.208-0.713 s, right temporal channels) with **AASP.** R^2: 0.7656, Est. Error: 0.0880

	Estimate	Est. Error	95% CI	BF_{10}
Intercept	-0.03	0.23	-0.48, 0.44	
Condition	-0.11	0.12	-0.36, 0.13	**0.0694**
AASP	-0.14	0.24	-0.61, 0.33	**0.0988**
Cond × AASP	0.04	0.12	-0.21, 0.27	**0.0445**

Table S8: Model output **entrainment power** (5.556 Hz, left occipital channels) with **AASP**.
R^2: 0.5219, Est. Error: 0.1515
